# Estimating the seroprevalence of SARS-CoV-2 antibodies: Understanding population-level immunity in Albania at the end of the Alpha variant wave

**DOI:** 10.7189/jogh.12.03054

**Published:** 2022-07-25

**Authors:** Fabian Cenko, Alban Ylli, Margarita Prifti, Erkena Shyti, Erina Lazri, Melissa J Perry, Genc Sulcebe

**Affiliations:** 1 Catholic University Our Lady of Good Counsel, Tirana, Albania; 2 Institute of Public Health, Tirana, Albania; 3 Research Unit of Immunology, Academy of Sciences of Albania, Tirana, Albania; 4 University of Medicine, Faculty of Medical Technical Sciences, Tirana, Albania; 5 Milken Institute School of Public Health, The George Washington University, Washington DC, USA; 6 University of Medicine, Tirana, Albania

The extent of a population’s overall immune response to SARS-CoV-2 is a critical indicator for determining the actual spread of COVID-19 infection. The level of specific immunity against COVID-19 will depend on the intensity of the virus circulation in the community, the extent of preventive measures in place, the number and size of the most susceptible population groups, and undoubtedly the vaccination rate and the effectiveness of the different vaccines being used [1].

Monitoring antibody levels against SARS-CoV-2 virus is practical and frequently used as a proxy to estimate the level of collective immunity in a population [2]. Many publications have described the rates of seroprevalence and the level of antibodies against the SARS-CoV-2 virus at different stages of the pandemic and in different countries. However, no data corresponding to the end of the Alpha variant wave in Albania has yet been published. A systematic review and meta-analysis of global SARS-CoV-2 antibody seroprevalence synthesized reports from 74 countries during 2020, but Albania was not among them, given the lack of previously published reports [3]. Additionally, only a few studies examined SARS-CoV-2 seroprevalence in the summer of 2021, when vaccination campaigns were well established in most countries worldwide and when the Alpha variant wave had declined before the Delta variant started gaining momentum.

We will summarize our experience in Albania by estimating the population immunity using the level of specific IgG anti-S1 SARS-CoV-2 antibodies in a randomly selected urban population of Albania in June-July 2021, corresponding to the end of the wave caused by the SARS-CoV-2 virus Alpha variant. We also estimated associations between SARS-CoV-2 immunity status and relevant factors.

We measured the anti-S1 antibodies of the SARS-CoV-2 virus spike protein in a sample of urban population individuals aged 18 to 70 years, randomly selected from Tirana and Berat’s primary health centres’ electronic registers from June to July 2021.

Serological testing of all blood samples was performed by an ELISA method using a commercially available diagnostic kit that determines IgG class antibodies anti-S1 protein of SARS-CoV-2 virus (IgG anti-S1-SARS-CoV-2 ELISA, Euroimmun, Luebeck, Germany).

The results were evaluated semiquantitatively by calculating the ratio of the sample’s optical density against the calibrator (Index Ratio or IR) as following the manufacturer's protocol. The IR values above 1.1 were considered positive, those between 0.8 to 1.1 as borderline, and those below 0.8 as negative. We only considered the serum samples above 1.1 as seropositive.

We interviewed participants using a standardized questionnaire that collected information on status of symptomatic infection, status of vaccination, type of vaccine (ie, CoronaVac, AstraZeneca, Sputnik V, and Pfizer-BioNTech), and the number of vaccine doses received. Other factors related to demography and health status were also collected and analysed.

Primary outcomes included seroconversion rate (applying the 1.1 Index Ratio threshold level) and serum levels of IgG anti-S1-SARS-CoV-2 antibodies. Differences in seroconversion rates and comparison of mean antibody levels for each category were measured in bivariate analyses using the student's *t* tests and one-way ANOVA for continuous variables and χ^2^ tests for categorical variables. Multivariate analyses were performed using linear regression models. The significance threshold level was 0.05 for all statistical tests. Categories of interest were defined according to demographic factors (age and sex) or immunity-related factors (previous infection status, severity of clinical status of infection, vaccination status, and type of vaccine). In this manuscript, we referred to vaccination and symptomatic infection as immunogenic events.

The ethical committee of the Albanian Academy of Sciences approved the study protocol (Project number 33-07-05-2020) and informed written consent was obtained from all participants.

**Figure:**
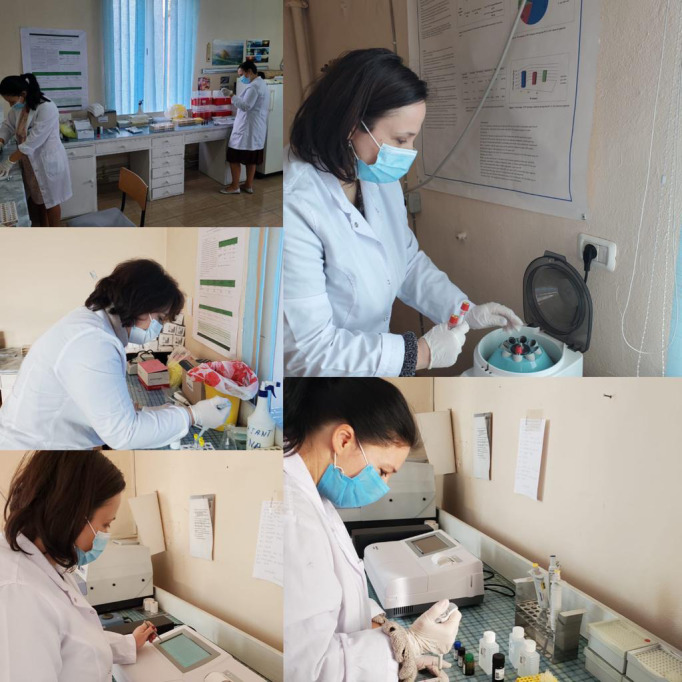
Photo: Research personnel processing participant blood samples in Tirana Albania. Source: personal photo archive, used with permission.

Our results showed that among 1527 study participants, 1120 (73.3%) had IgG levels above the IR threshold and were considered seropositive. The remaining 26% of the total sample with IgG levels below the IR threshold reported neither vaccination nor COVID-like symptoms. 49.3% of all the participants reported having had COVID-19 infection symptoms in the past and 41.8% said having been vaccinated with at least one vaccine dose. Infection and vaccination were found together in 17.3% of the population sample.

**Table 1** shows the seroconversion rate and serum level of IgG anti-S1-SARS-CoV-2 antibodies distributed according to several factors. Seropositivity and antibody level were highest among those who reported being vaccinated and had a previous infection, those who received a Pfizer-BNT162b2 vaccine, and those who had both vaccine doses. Seropositivity was also significantly higher among women and people over 60 years old. Among seropositive individuals who were not vaccinated, 42% did not report any symptoms.

**Table 1:** Seropositivity rate and levels of IgG anti-S1 SARS-CoV-2 antibodies in an Albanian community random sample (n = 1527) in July 2021.

Factor	Category	IgG anti-S1 SARS COV-2 seropositivity rates and the geometric mean of IR* values of IgG anti-S1 SARS-COV-2 antibodies					
		**Seropositive individuals (N)**	**%**	***P*-value†**	**Total individuals tested (N)**	**Geometric means of IR**	***P*-value†**
Age	18-60 y	686	68,9	0.000	996	3.5	0.000
	≥60 y	433	81,7		530	4.3	
	TOTAL	1119	73,3		1526	3.8	
Gender	Female	754	75.2	0.02	1002	3.9	0.012
	Male	366	69.7		525	3.5	
	TOTAL	1120	73.3		1527	3.8	
Immunogenic event	Only infection	376	76.9	0.002	489	3.3	0.000
	Only vaccine	321	85.8		374	4.5	
	Both	254	96.2		264	6.6	
	None	169	42.3		400	1.7	
	TOTAL	1120	73.3		1527	3.8	
COVID-19 Clinical manifestation	Mild	226	80.1	0.024	282	3.9	0.000
	Moderate	224	88.5		253	4.9	
	Severe	59	86.8		68	5.1	
	TOTAL	509	81.4		623	4.5	
Type of vaccines applied	CoronaVac	259	86.6	0.021	299	4.2	0.000
	AstraZeneca	127	91.4		139	6.1	
	Sputnik V	14	93.3		15	5.7	
	Pfizer-BNT162b2	174	95.1		183	6.9	
	TOTAL	574	90.3		636	5.4	
How many doses of vaccine	Only dose 1	100	78.1	0.000	128	4.7	0.002
	Both doses 1 + 2	450	93.6		481	5.6	
	TOTAL	550	90.3		609		

IR – index ratio, OD – optical density

*The IR was calculated as the ratio of the sample's OD to the OD of the cut-off value in the ELISA test.

†Using a one-way Analysis of Variance (ANOVA).

## DISCUSSION

The study results represent the first general population data about the Albanian population’s SARS-CoV-2 immunity level at the end of the Alpha variant wave. They also help characterize the factors that contributed to immunity status in this understudied population. These data contrast with prior seroprevalence information we have reported from one year earlier in June-July 2020, and in December 2020, before the Alpha variant surge in Albania; we found using the same methodology seroprevalence rates of 7.5% and 48.2%, respectively [4]. A recent study conducted among health care workers in Albania between February and May 2021 has found a seroprevalence of 72% [5].

The overall seroprevalence level of 73.3% found in June-July 2021 reflects the immunity status of the unselected urban Albanian population aged 18 to 70 years at the end of the Alpha variant wave and just before the consecutive Delta variant surge, which corresponded with a significant reduction in the incidence of COVID-19 infection in Albania. The pre-study period of April-June 2021 corresponded with a significant reduction in the incidence of COVID-19 infection in Albania. Still, it was the time when the country started a large-scale vaccination campaign with various vaccine types.

At that time, similar seroprevalence rates were reported in other adult populations, such as an average of 89% reported across testing centres in the USA [6], 66% in Geneva, Switzerland [7], and around 68% in the population over six years old in India [8]. There are no SARS-CoV-2 seroprevalence data reports during the summer of 2021 from other South-Eastern Europe or Balkan countries.

Our results show that the Albanian population’s immunity could be attributed predominately to past disease, as 56.3% of seropositive individuals reported a previous COVID-19 infection. Only 51.3% of them reported receiving at least one vaccine dose.

In Geneva, Switzerland, the seropositivity attributed to past infection was 29.9% in June-July 2021 [7]. At the same time interval in India, seropositivity attributed to past infection was more prevalent than from vaccination, considering that the seroprevalence among unvaccinated adults was 62.3% [8].

The seropositivity rate and serum antibody levels were significantly higher in vaccinated individuals than in those with past infections, which seems to be related to the shorter time from the vaccination (average time of 4.2 months) than the elapsed time since the SARS CoV-2 infection (average time of 7.3 months).

Interestingly, 42.3% of all individuals without reported infection or vaccination were seropositive. This result is similar to other studies with the same seroprevalence rates among symptomatic individuals [9].

Although IgG level was higher in women than men in the unadjusted analyses, this difference became irrelevant when multiple regression analyses were conducted (ie, vaccine type and covid-19 clinical presentation). The seropositivity rate and antibody levels were higher in those over 60 than in those under 60 years of age. This difference likely reflects a national policy recommendation targeting older age groups vaccination.

The seropositivity rate and antibody serum levels in vaccine-type categories were of particular interest in a country where different vaccines were used. The Pfizer-BNT162b2 vaccine showed the highest seropositivity rate and serology level, followed by the AstraZeneca and CoronaVac vaccines. Similar results have been reported in other studies [10]. The multivariable analyses revealed that this difference between different types of vaccines in our study sample is not influenced by the first or second dose, by the age of individuals under or above 60 years of age, or by having had a previous infection.

The study limitations include different response rates by gender, with males having a higher refusal rate than females (24% and 5%, respectively), and by age, with the youngest refusing the most. As the study sample is based on primary health care catchment populations, it most likely reflects Albania's health care user profile. Women are more inclined to use health care and are consequently more open than men to participate in a health survey. Also, there were only two cities included in the survey, and they may not fully represent the urban population in Albania. No data were collected for estimating the risk of breakthrough infections or re-infections amongst participants since that was not part of the study objectives.

Our results by age groups, gender, previous clinical infection, or vaccination status offer essential information for controlling COVID-19 through vaccination campaigns and behavioural changes. The two waves of data collection from 2020 also provide a unique opportunity for longer-term systematic monitoring of SARS-CoV-2 seroprevalence in Albania, and for a better understanding of population immunity patterns in the context of emerging new variants.
